# Supplementary material for: How does physical activity improve adolescent resilience? Serial indirect effects via self-efficacy and basic psychological needs
Source: PeerJ. 2024 Feb 29;12:e17059. doi: 10.7717/peerj.17059 (PMC10909365; doi:10.7717/peerj.17059)
Supplement: Supplemental Information 2 — An English-language codebook on the Research data [file peerj-12-17059-s002.docx]

An English-language codebook on the Research data

**1. exe-1~ exe-8 represent 8 items in the Physical Exercise Questionnaire. Physical Exercise Questionnaire includes Exercise adherence and exercise commitment subscales. Each item is scored on a 5-point Likert scale ranging from 1 (totally disagree) to 5 (totally agree).**

**(1) Exercise adherence**

exe-1: It is difficult for me to quit physical exercise

exe-2: If I do not exercise for a few days, I have a strong desire to participate in physical exercise

exe-3: It is difficult for me to accept a lifestyle of being lack of physical exercise

exe-4: Physical exercise is an indispensable part of my life

**(2) Exercise commitment**

exe-5: I insist on physical exercise well

exe-6: I often work by fits and starts on physical exercise

exe-7: I have the habit of exercising

exe-8: I often participate in sports activities

**2. eff-1~ eff-10 represent 10 items in the General Self-Efficacy Scale. Each item is scored on a 4-point Likert scale ranging from 1 (not at all sure) to 4 (completely true).**

eff-1: I can always manage to solve difficult problems if I try hard enough.

eff-2: If someone opposes me. I can find means

eff-3: It is easy for me to stick to my aims and accomplish my goals.

eff-4: I am confident that I could deal efficiently with unexpected events.

eff-5: Thanks to my resourcefulness, I know how to handle unforeseen situations.

eff-6: I can solve most problems if I invest the necessary effort.

eff-7: I can remain calm when facing difficulties because I can rely on my coping abilities.

eff-8: When I am confronted with a problem, I can usually find several solutions.

eff-9: If I am in a bind, 1 can usually think of something to do.

eff-10: No matter what comes my way, I’m usually able to handle it.

**3. psy-1~ psy-12 represent 12 items in the Basic Psychological Needs in Exercise Scale. Basic Psychological Needs in Exercise Scale contains three subscales, competence, relatedness, and autonomy. Each item is scored on a 7-point Likert scale ranging from 1 (strongly disagree) to 7 (strongly agree).**

**(1) Autonomy**

psy-3: We do things that are of interest to me

psy-6: I feel that the way PE is taught is the way I would like to

psy-9: I feel that the way classes are taught is a true expression of who I am

psy-12: I feel like the activities we are doing have been chosen by me

**(2) Competence**

psy-1: I feel that I improve even in the tasks considered difficult by most of the children

psy-4: I feel I perform correctly even the tasks considered difficult by most of the children

psy-7: I feel I do very well even in the tasks considered difficult by most of the children

psy-10: I am able to succeed even in the lessons considered difficult by most of the children

**(3) Relatedness**

psy-2: My relationships with my classmates are very friendly

psy-5: I feel like I have a close bond with my classmates

psy-8: I feel like a valued member of a group of close friends

psy-11: I feel like I belong to a large group of close friends

**4. res-1, res-2, res-4~ res-25 represent 24 items in the Connor-Davidson Resilience Scale. The scale contains tenacity, strength, and optimism subscales** (please note that the third item (res-3) has been removed due to low factor loading of less than 0.40). **Each item is scored on a 5-point Likert scale from 0 (never) to 4 (always)**

**(1) Optimism**

res-2: Close and secure relationships

res-4: Can deal with whatever comes

res-6: See the humorous side of things

**(2) Strength**

res-1: Able to adapt to change

res-5: Past success gives confidence for new challenge

res-7: Coping with stress strengthens

res-8: Tend to bounce back after illness or hardship

res-9: Things happen for a reason

res-10: Best effort no matter what

res-24: One works to attain one’s goals

res-25: Pride in your achievements

**(3) Tenacity**

res-11: One can achieve one’s goals

res-12: When things look hopeless, I don’t give up

res-13: Know where to get help

res-14: Under pressure, focus and think clearly

res-15: Prefer to take the lead in problem solving

res-16: Not easily discouraged by failure

res-17: Think of self as strong person

res-18: Make unpopular or difficult decisions

res-19: Can handle unpleasant feelings

res-20: Have to act on a hunch

res-21: Strong sense of purpose

res-22: In control of my life

res-23: I like challenge
