# Supplementary material for: How does physical activity improve adolescent resilience? Serial indirect effects via self-efficacy and basic psychological needs
Source: PeerJ. 2024 Feb 29;12:e17059. doi: 10.7717/peerj.17059 (PMC10909365; doi:10.7717/peerj.17059)
Supplement: Supplemental Information 3 [file peerj-12-17059-s003.doc]

STROBE Statement—Checklist of items that should be included in reports of ***cross-sectional studies***

|  | Item No | Recommendation |
| --- | --- | --- |
| **Title and abstract** | 1 | (a) How does physical activity improve adolescent resilience? serial Indirect effects via self-efficacy and basic psychological needs |
|  |  | (b) The present study provides novel theoretical insights into resilience in the field of sports psychology by establishing a connection between basic psychological needs and self-efficacy。 |
| Introduction | | |
| Background/rationale | 2 | In the context of challenging conditions such as interpersonal tension and severse academic pressure, adolescents are vulnerable to the harmful effects of negative events, which leads to a notable increase in anxiety, depression, stress levels, and feeling of loneliness. We suggest that certain students possess greater personal resilience, enabling them in adeptly managing adverse events. In other words, resilience plays an important role in explaining such variance. Personal resilience can be developed, reinforced, and sustained via physical activity (Ho et al., 2015). It is widely recognized that personal resilience can be developed, reinforced, and sustained through physical activity. Despite the abundance of research suggesting a positive relationship between physical activity and adolescent resilience, our understanding of the underlying psychological mechanisms remains limited. Thus, discerning how physical activity promotes teen resilience is vital. Recent studies showed that the relationship between physical activity and resilience could be mediated by basic psychological needs and self-efficacy. |
| Objectives | 3 | The main aim of this study was to investigate how physical activity improves adolescent resilience via serial indirect effects model using a cross- sectional approach with high school students. Specifically, we tested serial indirect effects of basic psychological needs and self-efficacy in the relationship between physical activity and resilience. To this end, we propose the following hypotheses: (H1) There is a direct positive association between physical activity and resilience; (H2) Physical activity has an indirect effect on resilience through satisfying basic psychological; (H3) Physical activity has an indirect effect on resilience through improving the levels of self-efficacy; (H4) Physical activity improves resilience through serial indirect effects of basic psychological needs and self-efficacy. |
| Methods | | |
| Study design | 4 | The current investigation employed a quantitative cross-sectional design, implemented on the ‘Wenjuanxing’ platform, to explore the direct and indirect associations between physical activity and resilience. Additionally, this study examined whether the association could be influenced by self-efficacy and basic psychological needs, using a serial indirect effect model. |
| Setting | 5 | A total of 1760 students were selected for our study over a two-week period in Sep. 2022. Written informed consent was obtained from all participants, which included a detailed introduction to the study, its purpose, as well as declarations of anonymity and confidentiality before their participation. |
| Participants | 6 | We utilized a simplified cluster sampling method to randomly select participants for our study. In our study, we firstly used a two-clusters design, consisting of Southern and Northern regions of China. We then randomly selected three high schools from each cluster, resulting in a total of six schools as selected clusters. From each of these schools, a certain number of students were then randomly selected from each school. Besides, we selected a wide range of centers to recruit participants, including both public and private centers. The participants completed an online questionnaire at their respective school. |
| Variables | 7 | Basic psychological needs, self-efficacy, physical activity levels, and resilience. |
| Data sources/ measurement | 8* | Physical activity was assessed using the Physical Activity Questionnaire (Wu 2016). It has been widely used to assess physical activity for Chinese adolescents (Li, Yu, & Yang, 2021). The Physical Activity Questionnaire is consisted of 8 items for assessing exercise adherence (i.e., 4-itemsp; e.g., “It is difficult for me to quit physical activity”) and exercise commitment (i.e., 4-itemsp; e.g., “I have the habit of exercising”).  The Basic psychological needs in exercise scale (BPNES), developed by Vlachopoulos, Katartzi, and Kontou (2011), was used to assess the Basic psychological needs. The Chinese version, which has been previously translated by Zhang (2015) was used in this study. This scale contained three subscales: competence, relatedness, and autonomy, consisting of 12-item.  General Self-Efficacy Scale (GSES) was adopted to measure individual self-efficacy. Specifically, we used a Chinese adaptation of GSES which has been validated to be used for Chinese adolescents (Zhang & Schwarzer, 1995). The scale consists of 10 items rating on a 4-point Likert scale from 1 (not at all sure) to 4 (exactly true).  A modified Chinese version of Connor-Davidson Resilience Scale was used in Chinese adults to asses resilience levels (Yu & Zhang, 2007; Li et al., 2021). The scale contains 25 items that evaluated tenacity (13 items), strength (8 items), and optimistic (4 items). |
| Bias | 9 | It is important to address the potential issue of common method variance, which arises from using self-reported data from the same source (Podsakoff et al., 2003). We employed Harman’s single factor test, utilizing un-rotated factor resolution (Podsakoff et al., 2003). The first factor alone explained only 37.29% of the variance in the data. |
| Study size | 10 | The full path model including physical activity level, resilience, basic psychological needs, and self-efficacy was assumed for the power analysis. The complete model involved four degrees of freedom, which needs a sample size of 1194 to identify a close fit of RMSEA value of 0.05 and 80% statistical power (Kim, 2005). A total of 1732 subjects were included in this study, which should provide sufficient statistical power. |
| Quantitative variables | 11 | This study does not involve groupings |
| Statistical methods | 12 | (a) The results of the present study were analyzed using a two-step process. Firstly, all preliminary data analyses were performed using SPSS 21.0. This included calculating descriptive statistics such as means and standard deviations for the social demographic and the variables to summarize the basic features of the data. Reliability analysis was then performed to explore the internal consistency of measurement scales. Pearson correlation analysis was then used to measure bivariate relationships among physical exercise, resilience, self-efficacy, and basic psychological needs. Finally, a hypothesized serial multiple mediator model was constructed using Amos 24.0 to investigate the direct and indirect effects of physical exercise on resilience. The goodness-of-fit indices was used to assess the fit of the serial multiple mediator model. To be specific, chi-square (χ2) test directly evaluates how well the proposed model fits the data (Bollen, 1989). If the χ2 result is significant (p < 0.05), it indicates that the proposed model does not fit the data. Conversely, a non-significant χ2 result (p > 0.05) indicates that the proposed model fits the data well (Barbeau, Boileau, Sarr, & Smith, 2019; Kim & Faith, 2020; Shah, 2012). The root mean square error of approximation (RMSEA) was also used to evaluate whether the proposed model supports factor structure (Fung et al, 2020; Lin, Imani, Griffiths, & Pakpour, 2021; Schweizer, 2010). The values less than 0.05 indicates a close model fit, while values between 0.05 and 0.08 indicate an acceptable model fit (Barbeau, Boileau, Sarr, & Smith, 2019; Browne & Cudeck, 1989). The Comparative Fit Index (CFI), normal fit index (NFI), goodness-of-fit index (GFI), and adjusted goodness-of-ft index (AGFI) were also used as important measurement indices, and a value of 0.95 or higher 0.90 was regarded as an acceptable model fit (Bentler, 1990). Overall, the fit of the proposed model was decided using chi-square RMSEA, CFI, NFI, GFI, and AGFI. Additionally, a bootstrapping analysis with 5000 random resamples was carried out to test the significance of the mediation effects and estimate 95% bias corrected bootstrap confidence intervals. The level of statistical significance for all indicators was set at p < 0.05. |
|  |  | (b) Excluding 28 participants due to regular answering patterns and faulty data, resulting in a valid response rate of 98.35%. |
| Results | | |
| Participants | 13* | (a) A total of 1732 students were included in the final sample after excluding 28 participants due to regular answering patterns and faulty data, resulting in a valid response rate of 98.35%. |
| Descriptive data | 14* | (a) The participants were in their second year of high school and ranged in age from 16 to 20 years (M = 16.51; SD= 0.77), with 47.63% of them being boys. With the exception of certain correlations related to sex, all of the main variables in the study were correlated, ranging from -0.338 to 0.703. |
| Outcome data | 15* | The mean value of basic psychological needs, self-efficacy, physical activity levels, and resilience is 24.04, 25.89, 62.84, and 62.37, respectively. |
| Main results | 16 | (a) The structural equation model was established using Amos software to explore a serial mediating effect of physical exercise on adolescents’ resilience between basic psychological needs and self-efficacy. The model’s fitting indexes were perfect, with χ2 /df = 6.848, RMSEA = 0.058, CFI=0.984, NFI =0.982, GFI=0.975, AGFI = 0.956. In serial mediation analysis, all the direct effects estimated from path analysis are shown in Fig 1. In this model, and independent of sex, the total effect of physical activity on resilience was found to be significant (β = 0.589, SE =0.025, p < 0.001, 95% CI [0.539, 0.635]). There was a statistically significant direct association between physical activity and resilience (β = 0.244, SE = 0.026, p < 0.001, 95% CI [0.194, 0.295]), which supports Hypothesis 1. Similarly, physical activity had a strong positive association with adolescents’ basic psychological needs (β= 0.424, SE = 0.029, p < 0.001, 95% CI [0.366, 0.480]) and self-efficacy (β= 0.244, SE = 0.030, p < 0.001, 95% CI [0.184, 0.302]). As can be seen there are also significant directs paths from basic psychological needs to resilience (β = 0.386, SE = 0.032, p < 0.001, 95% CI [0.324, 0.449]), self-efficacy to resilience (β = 0.384, SE = 0.032, p < 0.001, 95% CI [0.318, 0.446]). In a similar vein, basic psychological needs had a significant positive association with self-efficacy (β = 0.539, SE = 0.029, p < 0.001, 95% CI [0.480, 0.594]).  Next, significant indirect effects are presented for all paths of physical activity on resilience in Table 2. First, higher levels of physical activity were associated with higher levels of basic psychological need satisfaction and, in turn, to enhance resilience (β = 0.164, SE = 0.018, p < 0.001, 95% CI [0.132, 0.203]). The hypothesis 2 was supported. Second, a noteworthy indirect pathway was observed, indicating that physical activity exerted a significant influence on resilience solely through self-efficacy (β= 0.094, SE = 0.015, p < 0.001, 95% CI [0.067, 0.127]), providing support for Hypothesis 3. Finally, there was a significant indirect pathway for physical activity through basic psychological needs and self-efficacy in serial fashion (β= 0.094, SE = 0.015, p < 0.001, 95% CI [0.067, 0.127]); Great levels of physical activity was linked to the fulfillment of basic psychological needs and higher levels of self-efficacy and, in turn, ultimately contributing to the bolstering of resilience. Hence, Hypothesis 4 is also proved. |
| Other analyses | 17 | There is no other analysis |
| Discussion | | |
| Key results | 18 | Our results suggest that the effect of physical activity was associated with resilience through the following mechanisms: (i) indirectly via the satisfying of basic psychological needs; (ii) indirectly via higher levels of self-efficacy; (iii) indirectly via the fulfillment of basic psychological needs and, sequentially, increased self-efficacy. As expected, physical activity was related to resilience, but also indirectly via basic psychological needs, and indirectly self-efficacy. As well, engaging in physical activity was shown to contribute to increased levels of basic psychological needs, subsequently leading to greater self-efficacy and ultimately higher levels of resilience. |
| Limitations | 19 | Like other research, this study has several limitations that should be acknowledge. Firstly, although significant and positive relationships between various variables were observed, the cross-sectional design precludes establishing the exact causal relationships between them. Secondly, it should be noted that while this study investigated the link between basic psychological needs and physical activity, self-efficacy, and resilience, it did not address an issue whether autonomy, competence, and relatedness needs are independently important in the present settings. As suggested by Van der Kaap-Deederet al. (2017), each psychological need may contribute uniquely to adolescents’ well-being and may play a more distinct role in more specific domains. Therefore, future work should examine the association of each psychological need on the link between physical activity and resilience or self-efficacy. Thirdly, the present study also did not comprehensively estimate the association between the types, duration, intensity, and frequency of physical activity, so further empirical and qualitative work is necessary to determine their effects. Finally, the Physical Activity Questionnaire used in this study was specifically developed by Chinese researchers to assess physical activity behavior among the local population. Thus, it may be more suitable for the Chinese population due to its specific development within that context. |
| Interpretation | 20 | This is the first study to investigate physical activity, basic psychological needs, self-efficacy, and resilience as an interactive system. Our analysis has revealed a direct and significant correlation between physical activity and resilience among adolescents. We also found that the relationship between physical activity and resilience is influenced by the sequential associations between basic psychological needs and self-efficacy. Regular physical activity has the potential to induce positive psychology changes, ultimately contributing to the enhancement of resilience levels. Although future research is needed, our findings suggest that the fulfillment of basic psychological needs and higher levels of self-efficacy may play a pivotal role in improving resilience among adolescents within the context of physical activity environment |
| Generalisability | 21 | These findings provide the best support for the positive correlation between physical activity and resilience. Consequently, educators can design interventions promoting physical activity program tailored to individuals’ preferences and psychology needs. By cultivating an environment that encourages autonomy, relatedness, and competence, individuals are more likely to engage in physical activities willingly, thereby enhancing resilience. Additionally, therapists and counselors can incorporate physical activity into therapeutic interventions, recognizing its potential to not only improve physical health but also bolster emotional resilience. By integrating physical activities that align with individuals’ interests and psychological needs, therapy sessions can become more comprehensive and effective, promoting both physical and mental well-being. |
| Other information | | |
| Funding | 22 | No |

*Give information separately for exposed and unexposed groups.

**Note:** An Explanation and Elaboration article discusses each checklist item and gives methodological background and published examples of transparent reporting. The STROBE checklist is best used in conjunction with this article (freely available on the Web sites of PLoS Medicine at http://www.plosmedicine.org/, Annals of Internal Medicine at http://www.annals.org/, and Epidemiology at http://www.epidem.com/). Information on the STROBE Initiative is available at www.strobe-statement.org.
